# Advancing ethnobiology for the ecological transition and a more inclusive and just world: a comprehensive framework for the next 20 years

**DOI:** 10.1186/s13002-024-00661-4

**Published:** 2024-02-15

**Authors:** Ulysses Paulino Albuquerque, Alfred Maroyi, Ana H. Ladio, Andrea Pieroni, Arshad Mehmood Abbasi, Bárbara Arias Toledo, Farid Dahdouh-Guebas, Gustavo Hallwass, Gustavo Taboada Soldati, Guillaume Odonne, Ina Vandebroek, Joan Vallès, Julio Alberto Hurrell, Manuel Pardo de Santayana, María de los Ángeles La Torre-Cuadros, María Teresa Pulido Silva, Michelle Cristine Medeiros Jacob, Viviane Stern da Fonseca-Kruel, Washington Soares Ferreira Júnior

**Affiliations:** 1 Centro de Biociências, Cidade Universitária, Universidade Federal de Pernambuco, Recife, Pernambuco Brazil; 2 Department of Botany, University of Fort Hare, Private Bag X1314, Alice, 5700 South Africa; 3 Grupo de Etnobiología, INIBIOMA (Instituto de Investigaciones en Biodiversidad y Medio Ambiente, Universidad del Comahue- CONICET), Quintral 1250, S.C. de Bariloche, Rio Negro, Argentina; 4 University of Gastronomic Sciences, Piazza Vittorio Emanuele II 9, 12042 Pollenzo, Cuneo Italy; 5 Department of Medical Analysis, Tishk International University, Erbil, Kurdistan 44001 Iraq; 6 Department of Environmental Sciences, COMSATS University Islamabad, Abbottabad Campus, Abbottabad, 22060 Pakistan; 7 Instituto Multidisciplinario de Biología Vegetal (IMBIV-CONICET-UNC), Edificio de Investigaciones Biológicas y Tecnológicas, Ciudad Universitaria, Córdoba, Argentina; 8 Systems Ecology and Resource Management Research Unit (SERM), Department of Organism Biology, Université Libre de Bruxelles - ULB, Brussels, Belgium; 9 Ecology and Biodiversity, Laboratory of Plant Biology and Nature Management, Biology Department, Vrije Universiteit Brussel - VUB, Brussels, Belgium; 10 Mangrove Specialist Group (MSG), Species Survival Commission (SSC), International Union for the Conservation of Nature (IUCN), Zoological Society of London, London, UK; 11 Interfaculty Institute of Social-Ecological Transitions, Université Libre de Bruxelles - ULB, Brussels, Belgium; 12 Programa de Pós-Graduação em Ecologia Aplicada, Instituto de Ciência, Tecnologia e Inovação, São Sebastião do Paraíso, Universidade Federal de Lavras, Lavras, Minas Gerais Brazil; 13 Instituto de Ciências Biológicas, Universidade Federal de Juiz de Fora, Juiz de Fora, Minas Gerais Brazil; 14 Laboratoire Ecologie, Evolution, Interactions Des Systèmes Amazoniens (LEEISA), CNRS-Université de Guyane-IFREMER, 97300 Cayenne, French Guiana; 15 Department of Life Sciences and Natural Products Institute, Faculty of Science and Technology, University of the West Indies Mona, Mona, Jamaica; 16 Institute of Economic Botany, The New York Botanical Garden, Bronx, NY USA; 17 The Graduate Center, City University of New York, New York, NY USA; 18 Laboratori de Botànica, Facultat de Farmàcia I Ciències de L’Alimentació, Universitat de Barcelona, Av. Joan XXIII 27-31, 08028 Barcelona, Catalonia Spain; 19 Institut d’Estudis Catalans, C. Carme 47, 08001 Barcelona, Catalonia Spain; 20 Laboratorio de Etnobotánica y Botánica Aplicada (LEBA), Facultad de Ciencias Naturales y Museo, Universidad Nacional de La Plata, Investigador CONICET, Buenos Aires, Argentina; 21 Departamento de Biología (Botánica), Universidad Autónoma de Madrid, C/Darwin 2, Campus de Cantoblanco, 28049 Madrid, Spain; 22 Centro de Investigación en Biodiversidad y Cambio Global (CIBC-UAM), Universidad Autónoma de Madrid, Madrid, Spain; 23 Universidad Científica del Sur, Av. Panamericana Sur Km 19, Villa EL Salvador, 15067 Lima, Peru; 24 Grupo Sistemas Socioecológicos y Servicios Ecosistémicos, Facultad de Ciencias Forestales, Universidad Nacional Agraria La Molina (UNALM), Av. La Universidad S/N Lima 12, Lima, Peru; 25 Laboratorio de Etnobiología, Centro de Investigaciones Biológicas, Universidad Autónoma del Estado de Hidalgo, Cd. Universitaria, Carr. Pachuca-Tulancingo, Km 4.5 S/N, CP 42184 Pachuca, Hidalgo Mexico; 26 Laboratório Horta Comunitária Nutrir, Universidade Federal do Rio Grande do Norte, Natal, Rio Grande do Norte Brazil; 27 Jardim Botânico Do Rio de Janeiro, Diretoria de Pesquisas, R. Pacheco Leão 915, Rio de Janeiro, 22460-030 Brazil; 28 Universidade de Pernambuco, Campus Petrolina, BR 203, Km 2, S/N, Vila Eduardo, Petrolina, Brazil

**Keywords:** Indigenous Peoples and Local Communities (IPLC), Afro-descendant and other Marginalized, Minority, and Minoritized Communities (AMMC), Biocultural conservation, Biocultural diversity, Political, Ethical dimensions

## Abstract

This opinion piece, written by ethnobiologists from different parts of the world, emphasizes the importance of ethnobiology research in advancing contemporary biology, natural resource management, biodiversity conservation, sustainable development, and, especially, contributing to the ecological transition and more just and inclusive world. To achieve these goals, it is essential to develop research and collaborate with social groups that live in close relationship with nature in research activities, such as Indigenous Peoples and Local Communities (IPLC), as well as Afro-descendants and other Marginalized, Minority or Minoritized Communities (AMMC). Ethnobiology can identify and provide locally appropriate solutions to local problems, enabling sustainable resource management at the landscape level. The text explores important aspects that need to be considered to guide the future of ethnobiology in the next 20 years, aiming to integrate and amplify previous discussions held in the discipline and identify points that demand ongoing attention. This paper highlights reflections from diverse researchers, emphasizing how ethnobiology can embrace different perspectives and employ rigorous analysis of complex phenomena toward effective policies and practices. This approach holds the potential to address the challenges the planet is currently facing in the coming decades.

## Introduction

Ethnobiology, an interdisciplinary science studying the interrelationships between people and biota, has explored various scenarios related to human interactions with nature. In recent decades, researchers in the field have reflected on different theoretical, methodological, practical, including ethical and political dimensions associated with diverse human groups, particularly Indigenous Peoples and Local Communities (IPLCs),^1^^,^^2^ including Afro-descendants and other Marginalized, Minority, or Minoritized Communities (AMMC), with a recent focus on decolonization [4, 6]. These reflections are essential for guiding actions and addressing challenges such as climate change, biocultural conservation, food security, and sovereignty.

**Fig. 1:**
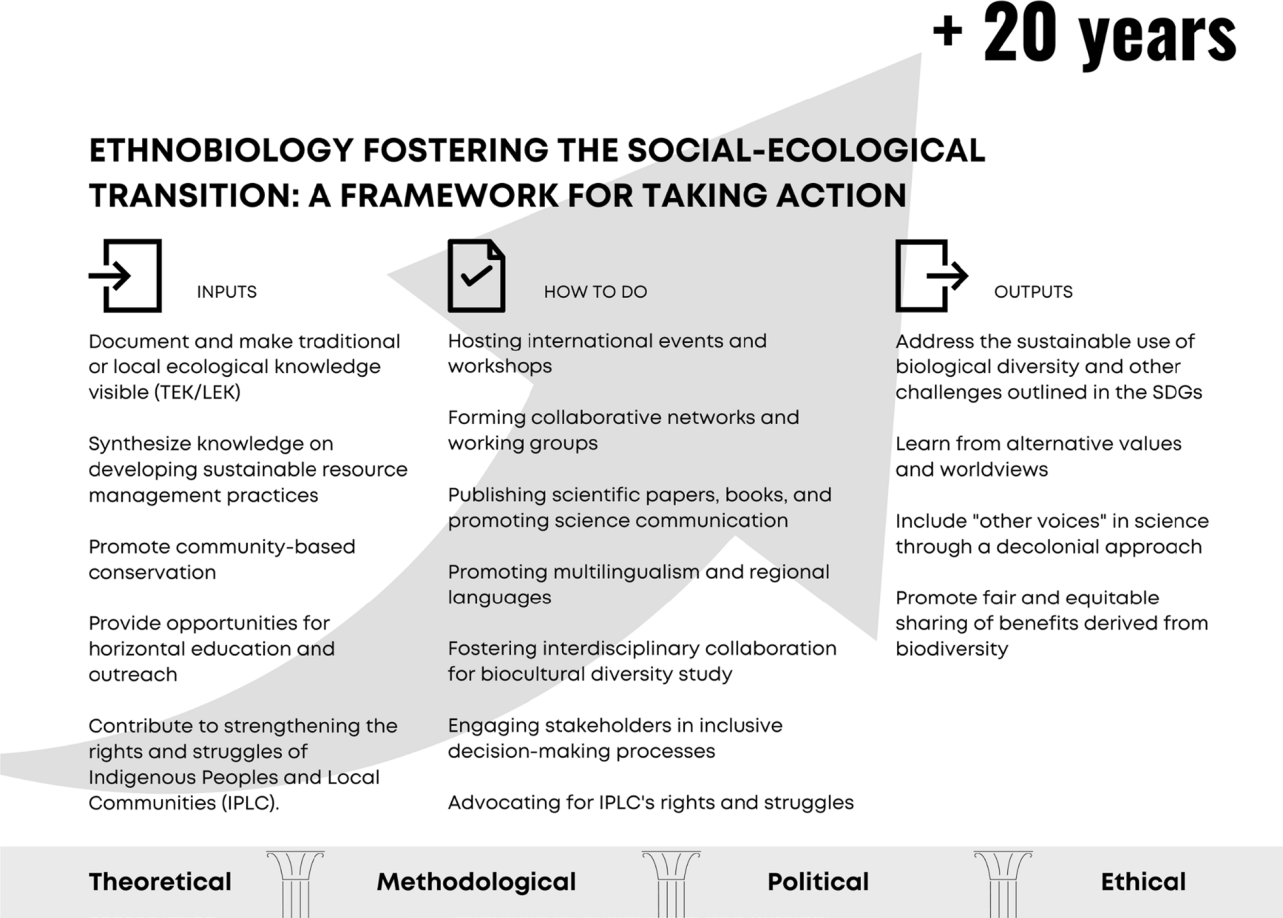
A framework for taking action in social-ecological transition within ethnobiology with suggestions for inputs, expected outputs, and practical actions (i.e., how to do it). The framework is based on the foundation of categories of thinking required to successfully navigate the ecological transition toward sustainability

This text presents the collective reflections of researchers on the future of ethnobiology in the next 20 years. Addressing the complex challenges of human–nature relationships, these reflections are important for proposing integrated agendas encompassing theoretical, practical, sociopolitical, environmental, and ethical aspects (Fig. 1). Through the reflections presented in this text, we can initiate a discussion for establishing a robust ethnobiology for the next decades, enhancing its potential to make a significant impact by understanding and actively engaging with the diverse dimensions of human–nature relationships.

## An ethnobiology for the next 20 years

This article reflects on the future of ethnobiology over the next 20 years, emphasizing the immediacy of the actions discussed. While the 20-year framework provides a long-term perspective, it is not a timeline for deferring urgent measures. Each strategy, although set against a backdrop of long-term planning, is driven by current, pressing challenges that require immediate attention. The time frame is intended for ongoing evaluation and adaptation of our responses, not for delaying their initiation. This approach underlines the necessity of proactive and responsive actions in ethnobiology, calling for immediate implementation even as we prepare for future challenges and opportunities.

The ethnobiological approach is central to advancing contemporary biology and natural resource management, biodiversity conservation, and sustainable development. In order to achieve these goals, it is essential to develop research with IPLC, using culturally appropriate and collaborative approaches capable of expanding their rights, especially the right to territory, right of access to biodiversity, and the right to consultation, as well as the voices of AMMC [1, 7]. These social groups historically live in a close and strong relationship with nature, where they developed their social and cultural systems. IPLC and AMMC have long been building, demanding, and fighting for transformative change in the face of perpetuated social-ecological injustices and drastic environmental and social deterioration. For this reason, several authors have considered that various aspects of IPLC and AMMC practices and positioning may be leveraged to bring about transformative change thinking [8, 9]. Ethnobiology can help to identify and spearhead locally appropriate solutions to local problems and enable sustainable resource management at the landscape level [1, 8–11].

These cultural systems can be based on self-sufficiency and rooted in non-exploitative relational models [12] with an ethical commitment to renewing natural cycles. Since ancient times, these social groups have established a close and deeply emotional relationship with nature, transmitting their dependency and ethics of care for nature from generation to generation [13–15]. Ethnobiology, as a scientific discipline committed to the necessary struggle for a socially and ecologically just society, has a crucial role in following the proposal of making visible the ideas and actions of indigenous and marginalized peoples. In addition to making visible traditional systems of knowledge and management of biodiversity, it is necessary to demonstrate scientifically that these are more sustainable and efficient, creating strategies to incorporate these into public policies. Thus, in situations where the sustainability of traditional management systems is compromised, usually due to threats and strangulation of traditional territories, Ethnobiology must be a tool to find new alternatives for use, assuming that access to biodiversity by these peoples is a human right. Because in the case of the environmental crisis, the omission of the IPLC thinking is not only not inclusive, but it also narrows the horizons of the search for possible solutions and the multicultural understanding of natural and social phenomena [16].

It is imperative to underscore the paramount importance of inclusivity in ethnobiology, mainly by providing and strengthening space for “other” voices, including minority groups, women, scholars, and activists from the global south. Research in ethnobiology should prioritize collaborative and intercultural research, seeking the perspectives of local communities, registering and relating knowledge in the local languages, and integrating it with meanings and local cultures. This inclusive approach enables the incorporation of non-North-American-Eurocentric perspectives and fosters the exploration of new conceptualizations and innovative participatory methods. Furthermore, it is recommended that research agencies and funding bodies establish specific measures to facilitate global south scholars in studying communities and regions within the “global north.” This proactive step would yield fresh inspirations and insights, addressing a significant concern in ethnobiology: the historical imbalance perpetuated by colonialism, wherein global south scholars predominantly have to focus on studying their communities. In contrast, scholars from the global north can examine diverse cultures from their own region and around the globe.

Historically, systemic obstacles such as unequal access to higher education and the marginalization of perspectives in predominantly non-Indigenous academic environments have contributed to the underrepresentation of Indigenous, Afro-descendant, and other minoritized local community scholars in ethnobiology. Recognizing this, there is a pressing need to create more inclusive spaces within the field that genuinely value and incorporate diverse methodologies and viewpoints. This involves re-evaluating current research practices, fostering equitable collaborations, and ensuring that these communities have a significant role in shaping research agendas. By committing to these changes, we aim to diversify the voices in ethnobiology, thereby enriching the field with a broader range of insights and approaches. Our acknowledgment and active efforts toward inclusivity and representation are crucial steps in addressing this challenge.

It should be emphasized that IPLC and global south researchers have suffered from a history of exclusion and monologue, with the imposition of a single language as the means of communication of the hegemonic science, English. Fortunately, for some time now, a new phase has been developing in which interculturality is central [17–19]. However, only when multilingualism becomes part of our scientific exchange will it be possible to work on equal terms without power asymmetries. Historically, ethnobiology has focused on a linguistic perspective to assess how diverse human groups name different elements in nature, revealing the vast linguistic diversity across the globe. These studies are essential for developing a multilingualism perspective, particularly by acknowledging that languages (especially minoritized ones, in underdeveloped, developing, or developed areas) are as significant as biodiversity in ethnobiological terms (see [20]).

The priority for ethnobiology is (a) to secure a position in the political and institutional sphere of countries, particularly the poorest, or (b) technically support the IPLC in occupying these positions. That is, to create *ex professo* and long-term institutions that can promote the sustainable rural development of communities. The aim is not to replicate a vision of handouts but to accompany the communities in their development and self-management based on an emic approach. Moreover, the ethnobiologist needs to assume positions of political power within his or her country. Public policy with an ethnobiological vision is essential, particularly in developing countries. The political and institutional consolidation of ethnobiology in each of these countries would provide an important counterweight to the political ups and downs that have generally ended with a stroke of the pen for institutions related to ethnobiology. This was the case in Mexico for the Instituto Nacional para el Estudio de las Plantas Medicinales Mexicanas and the Instituto Nacional de Investigaciones sobre Recursos Bióticos. Both no longer exist.

There are several initiatives in this sense, but we can emphasize research carried out with indigenous peoples in the Northwest Amazon (Brazil). Intercultural and collaborative research has been key to Amazon’s environmental, climate, and sustainability governance [21]. We know that several Amazonian landscapes are products of the management and interaction between indigenous societies and their environment [22]. However, until recently, local knowledge did not circulate in writing (only orally and from generation to generation). Currently, local knowledge, both every day and ancestral, is being synthesized and reinvented in intercultural and collaborative research, constituting new knowledge that values local knowledge and addresses the processes of formulating environmental policies for the Amazon. Important products have been developed through the collaboration of more than two decades between researchers from the Instituto Socioambiental (www.socioambiental.org) and the Federation of Indigenous Organizations of Rio Negro (http://www.foirn.org.br). As a result of this collaboration, a network of non-indigenous and indigenous researchers (especially from the Tukano indigenous people) was started to document their knowledge in an integrated way, such as the Tukano ecological calendar (with records of the cycles of fish, amphibians, birds, mammals, insects, plants, daily work in agriculture, fishing, gathering and hunting, rituals, disease prevention, and cure, diet, and behavior) [23].

Developing a robust theoretical and methodological framework is crucial for the evolution of ethnobiology. Achieving this requires ongoing debate and discussion to establish complex models and ensure a comprehensive understanding of complex phenomena. The theory is the foundation for developing ethnobiology, while methodology provides the tools to generate diverse models that move away from reductionism. This approach ensures that the complexity of the phenomena under study is adequately addressed and understood. Ethnobiology can and should also be integrated into the study of social-ecological systems [24, 25].

Moreover, theories and methods have to leave space for “other” visions, which are still largely dominating Western ethnobiology. It also requires the triangulation of cohesive methods that integrate various modes of inquiry, including field-based research, quantitative data analysis, participatory survey techniques, as well as non-intrusive documentation techniques (for the latter, see [26]). Especially the new trajectory traced by the European Union as a new possible horizon in science and regarding a truly “citizen science” approach could be crucial: methods should take into account the possibility of including not only the local communities (what nowadays only still partially happens), but also simple citizens as co-designers and participating actors within the research platforms. Ethnobiological research bridges the gap between several social groups, including minorities and the scientific community. Thus, ethnobiology can support effective public policies that ensure the livelihoods of different groups by reducing social inequalities. In addition, these actions will enhance the capacity of ethnobiologists and contribute to the preservation of biological and cultural diversity on a global scale, especially by fostering the adaptability and resilience of the social-ecological systems [27].

In this sense, it is necessary to emphasize comprehensive documentation to strengthen ethnobiological research’s theoretical and methodological perspectives. This documentation should encompass philosophical and procedural aspects and dimensions such as biology, anthropology, socioeconomics, and culture. Moreover, it is vital to incorporate this documentation into educational curricula at all levels and provide appropriate training and skills development opportunities for young researchers, particularly in developing and underdeveloped regions [28]. Financial assistance is also crucial in supporting field-based training and research activities in ethnobiology. It is imperative that all stakeholders, including government agencies, non-governmental organizations, policymakers, media coordinators, and community representatives, recognize and promote the significance of ethnobiological studies in socio-economic development and the conservation of natural resources and biocultural heritage. Working to promote the respect and consideration of local, indigenous, or traditional perspectives, experiences, and knowledge among different stakeholders allows for integrating research methods and approaches, identifying areas of convergence, and pursuing collaborative solutions.

A new era in ethnobiology is needed to highlight the value of the work currently being done in the discipline, and its contribution to sustainable development, ecological transition, and a more just and inclusive world. For example, ethnobiology contributions to mitigating climate change or enhancing the SDGs (https://sdgs.un.org/goals) have been invisible. This subalternation is partly due to the lack of robust and well-thought theoretical frames of our research paths and findings. However, other factors are related to power asymmetries between environmental and social disciplines—as well as the isolation from each other of the groups of scientists practicing them—that leave ethnobiology in a sometimes discredited and misunderstood place due to its peculiarly multidimensional and multifaceted nature.

Ethnobiology can especially play a quintessential role in implementing the so-called “ecological transition” that the world is facing in the next century by:

1. Documenting and making visible TEK/LEK: Ethnobiologists can collaborate with indigenous and local communities to document their knowledge and practices related to sustainable resource use and conservation; these inspirations contribute to making traditional knowledge and management more visible and capable of developing more effective conservation strategies and to inform policy decisions related to resource management.
2. Synthesizing knowledge on sustainable resource management practices: Ethnobiologists can work with communities to develop durable resource management practices that are based on local knowledge and practices that could include the development of agroforestry and agroecological systems, sustainable harvesting techniques, and other approaches that promote long-term sustainability. In some situations, the use of biodiversity by IPLCs is still criticized by science, such as conservation biology. Thus, ethnobiology must produce data on the real ecological implications of traditional use and, when this is not sustainable, contribute to guaranteeing the right to use and access biodiversity.
3. Promoting community-based conservation: Ethnobiologists should work with communities to develop and implement community-based conservation programs based on local wisdom that could help promote the conservation of biodiversity and ecosystems while supporting local communities’ livelihoods and cultural practices.
4. Building an international community of belonging: Accelerating discussions within ethnobiology about diverse scholarly perspectives and opinions in the field, including constructive discussions around ethics, inclusion, and action-oriented research, or resolving disagreements in terminology and sharing case studies and research priorities from different geographies to learn from each other and broaden our inner horizons.
5. Providing horizontal education and outreach: Ethnobiologists could offer education and outreach to the public and policymakers about the importance of TEK/LEK; this could build support for conservation and sustainability initiatives and promote a greater understanding of the links between human well-being and ecological health.
6. Contributing to the comprehension of variations in the perception of natural resources, land tenure, food security, and livelihoods among communities, especially indigenous communities, in assessing and analyzing the extent of the exercise of acquired collective rights [29]. This holds significant value in making informed decisions regarding conservation efforts, collaborating with local groups, developing national interest projects, and formulating future scenarios [30].
7. Contributing opportunities to strengthen the rights and struggles of IPLCs: Ethnobiologists must produce, in full partnership with IPLCs, research that strengthens their rights, such as the access and use of biodiversity, the right to self-recognition, the right to free and informed prior consultation, the right to maintain traditional ways of life and the right to territory. This research can develop theoretical syntheses about the struggle strategies developed by the IPLCs throughout the centuries. In addition, it is necessary to ensure the presence of IPLCs in government structures and guarantee technical support, such as translating legislation and international agreements. In this regard, it is important to respect the different values, times, and political organization of the IPLCs. It will be important here that ethnobiologists advocate that IPLCs are not monoliths and that scholars do not inadvertently perpetuate the exclusion of some communities because they do not neatly fit internationally accepted definitions.

Individuals and institutions around the world have already made significant contributions to the seven aspects we have raised, particularly the work of the International Society of Ethnobiology (https://www.ethnobiology.net). However, we are unaware of these strategies as a collective agenda for the global community of ethnobiologists.

The next 20 years will be crucial for ethnobiology, and the main task should be to develop a comprehensive and cohesive approach that integrates applied and political aspects. This requires a diligent effort to document and analyze the processes through which TEK/LEK is revalued and integrated into global and local ecological transitions and community well-being. To succeed in this endeavor, it is crucial to focus on understanding the co-creation and design of these processes and their impacts on societies and communities while identifying and addressing the bottlenecks that impede their implementation. Especially the “making” of these processes will have to be considered a new focus in the ethnobiological scientific outputs, overcoming the idea that “applying” research is beyond the scientific community’s interest. At the same time, in its essence, the contrary should be true. In a nutshell, we need more papers narrating these difficult implementation processes, underlying those factors and circumstances which influence the development of successful stories and failures in applying ethnobiological data. In addition, a crucial priority for Ethnobiology is to develop a theoretical, conceptual, and methodological framework that supports the development of skills and encourages studies that promote the political empowerment of the local communities and more symmetry between TEK and academic knowledge. This involves making IPLCs and AMMC visible, strengthening their rights [31], and striving toward a more just society that eliminates the exploitation of humans and nature. Additionally, it is necessary to address power dynamics and inequalities that hinder the recognition of traditional knowledge and management practices.

Ethnobiology can significantly contribute to understanding how cultural and environmental factors influence food and nutrition security at the system level. To actively participate in the global debate on food security, ethnobiology researchers need to develop macro-scale analyses and establish methodological strategies for comparing research data from different regions. Furthermore, improving data-gathering, more creative dissemination protocols, and good-practices-guidelines could be essential. Collaboration among researchers from diverse professional and geographic backgrounds is crucial in achieving these objectives.

Between 60 and 80% of food production in developing countries is in the hands of rural women [32]. Unfortunately, in many developing and underdeveloped countries, stakeholders and policymakers frequently ignore and underestimate women’s IPLC. Refraining from considering women’s knowledge renders all efforts toward managing, conserving, and sustainable utilization of natural resources meaningless [33]. The activities assigned to women involve collecting wild foods and medicines and/or caring for plants (herbaceous and woody) and/or domesticated animals, carrying water and firewood, preparing and selecting meals, performing health care activities, and storing food and medicines, among others. Women have a long history of learning caregiving tasks and spend many hours daily in domestic, agricultural, and reproductive work. Such roles are often naturalized, unpaid, and under-recognized but mainly expose women to daily and direct contact with environmental pollution, resource scarcity, and climate change [32, 34]. It is necessary to encourage an ethnobiology that is critical of gender asymmetries. At the same time, it is necessary to open new perspectives that allow for the consideration of the complex gendered dimension and biases of research and practice without old stereotypes, an aspect still obscured in ethnobiological scientific production, especially looking at the non-binary dimensions of gender.

Ethnobiology should embrace a multidisciplinary and trans-disciplinary approach and foster collaboration with diverse scientific fields and political actors. Doing so can effectively address pressing global challenges such as health disorders, climate change, food insecurity, and natural resource management. Ethnobiological research holds the potential to provide valuable insights from IPLC and AMMC knowledge systems, contributing to the development of solutions in fields such as pharmaceuticals, biomedicine, agriculture, and sustainable resource management. Moreover, to enforce their access rights and intellectual property rights to their knowledge in compliance with all international standards. In a multidisciplinary approach, theoretical and methodological rigor must go hand in hand with guaranteeing ethical and solidary work with communities.

In the face of critical challenges such as climate change, biodiversity loss, and the conversion of natural habitats, Ethnobiology emerges as a discipline capable of modeling, interpreting, and establishing productive dialogs among IPCLs, scientists, and government. It holds the potential to bridge traditional forms of production with public policies, foster authentic and enriching debates, and showcase the diversity of approaches to nature. Ethnobiology makes scientifically public the demands, knowledge, and strategies of adaptation of social groups, often invisible to the public power and most of the population. Additionally, Ethnobiology ideally describes and raises awareness about urgent issues more intensively, developing proposals and providing or attempting to provide possible solutions [35, 36]. For example, ethnobiologists must proactively combat epistemic and social injustices and ensure that their questions, methodologies, and interpretation of results remain uninfluenced by hegemonic practices [4].

## Proposals for a future agenda

To foster discussions in the scientific community on the topic of ethnobiology in the next 20 years, the following proposals can be implemented:

- Organizing international and trans-regional conferences and symposia focused on ethnobiology, inviting renowned experts, emerging researchers, and representatives of marginalized groups and local and traditional communities. These events can provide spaces for research presentations and discussions on theory and methodology and address political issues and emerging challenges. Also, opening our events to traditional ways of communication through more sensorial channels (music, poetry, exhibitions of objects, traditional art, for example) is a way to bridge the gap between the Scientific and TEK language.
- Establishing thematic working groups that address specific issues addressed here, bringing together researchers from different institutions and regions to share knowledge, exchange experiences, and develop collaborations.
- Encouraging researchers to publish scientific articles and books and disseminating tools devoted to laypeople that delve further into the themes discussed here, thus providing a solid foundation for discussions and fostering new approaches and perspectives.
- Encourage the publication of texts in scientific journals on the experiences of social movements related to the themes of ethnobiology. Social movements develop different activities and understandings related to biodiversity; however, these experiences, in most cases, need to be solidified in academic texts, which limits the socialization of popular experiences and accumulations.
- Establishing collaborative networks among researchers, institutions, and IPLC and AMMC, facilitated by online platforms that enable effective communication, resource sharing, and coordination of research projects. In particular, it recognizes the noteworthy accomplishment of scientists from less affluent nations who are doing great ethnobiology. They are doing more with less.
- Organizing workshops and training sessions that specifically address the practical aspects of ethnobiology, empowering researchers to effectively tackle the challenges identified in this text.
- Embracing multilingualism and regional and local languages in ethnobiology, both within the international research arena and also at local level. This will allow our discipline to give a better voice to IPLC and AMMC worldwide (see [37]).
- Encouraging collaboration between ethnobiologists and researchers from various fields (such as social anthropologists, human geographers, linguists, botanists, zoologists, and agronomists) to enrich ethnobiological research, integrate different perspectives, and address global challenges.
- Supporting scientists to present their science-based recommendations in a way that empowers non-technical decision-makers in the political environment. In this sense, training environmental agents and managers are essential to recognize and value social-ecological systems and support effective public policies.
- Strengthening constant thinking, operating in work teams, and sharing datasets and reflections based on appropriate ethical frames and a clear anti-racist, anti-patriarchal, anti-colonialist, anti-heteronormative ethos can enhance equality and justice (see [38]).
- Contribute to strengthening the rights and struggles of the IPLCs, whether through scientific development or support for organized social movements and the occupation of government decision-making spaces.
- Exploring biocultural diversity across various spatial and temporal scales, including diverse terrestrial and aquatic environments (high mountains and arid regions, including desert and semi-desertic areas, tropical forests, high-latitude arctic regions, isolated tropical islands) (see, for example [39]), especially within developing and emerging countries is paramount.

These proposals contribute to the promotion of rich and in-depth discussions in the scientific community about ethnobiology, stimulating theoretical, methodological, and practical advancements in this field of study and promoting the integration of traditional/local knowledge into both broader socio-environmental issues and the real-life we citizens/hosts on this planet.

## Data Availability

Not applicable.

## Abbreviations

IPLC: Indigenous Peoples and Local Communities
AMMC: Afro-descendants and other Marginalized, Minority or Minoritized Communities
LEK: Local Ecological Knowledge
TEK: Traditional Ecological Knowledge

## Footnotes

1 We understand that Indigenous Peoples and Local Communities (IPLCs) represent diverse groups. Based on the International Labour Organization’s Convention 169, Indigenous Peoples are defined as those who “descend from the populations which inhabited the country, or a geographical region to which the country belongs, at the time of conquest or colonization or the establishment of present state boundaries and who, irrespective of their legal status, retain some or all of their own social, economic, cultural and political institutions,” self-identifying as Indigenous. Additionally, the Convention 169 highlights that groups of people who recognize themselves in ways that “social, cultural and economic conditions distinguish them from other sections of the national community, and whose status is regulated wholly or partially by their customs or traditions or by special laws or regulations” are considered Local Communities. Notably, these groups have significant and complex interactions with their environments to maintain their subsistence and cultural practices [1–3]. We also understand that IPLC should encompass groups that have migrated to different environments and descendants of enslaved peoples who have been forcibly removed from their places of origin and currently establish relationships with environments in other areas, as well as other minoritized communities, which suggests an ongoing reflection on what we understand by “local” [4].

2 We have opted to capitalize Indigenous and other Indigenous-centered terms in accordance with Gregory Younging’s book [5].
